# Deciphering Target Protein Cascade in *Salmonella typhi* Biofilm using Genomic Data Mining, and Protein-protein Interaction

**DOI:** 10.2174/1389202924666230815144126

**Published:** 2023-10-27

**Authors:** Aditya Upadhyay, Dharm Pal, Awanish Kumar

**Affiliations:** 1 Department of Biotechnology, National Institute of Technology Raipur, Raipur, 492010 (CG), India;; 2 Department of Chemical Engineering, National Institute of Technology Raipur, Raipur, 492010 (CG), India

**Keywords:** *Salmonella typhi*, biofilm, antibiotic resistance, drug targets identification, anti-biofilm therapeutics, typhoid fever

## Abstract

**Background:**

*Salmonella typhi* biofilm confers a serious public health issue for lengthy periods and the rise in antibiotic resistance and death rate. Biofilm generation has rendered even the most potent antibiotics ineffective in controlling the illness, and the *S*. *typhi* outbreak has turned into a fatal disease typhoid. *S. typhi* infection has also been connected to other deadly illnesses, such as a gall bladder cancer. The virulence of this disease is due to the interaction of numerous genes and proteins of *S*. *typhi*.

**Objective:**

The study aimed to identify a cascade of target proteins in *S. typhi* biofilm condition with the help of genomic data mining and protein-protein interaction analysis.

**Methods:**

The goal of this study was to notice some important pharmacological targets in *S. typhi.* using genomic data mining, and protein-protein interaction approaches were used so that new drugs could be developed to combat the disease.

**Results:**

In this study, we identified 15 potential target proteins that are critical for *S. typhi* biofilm growth and maturation. Three proteins, CsgD, AdrA, and BcsA, were deciphered with their significant role in the synthesis of cellulose, a critical component of biofilm's extracellular matrix. The CsgD protein was also shown to have high interconnectedness and strong interactions with other important target proteins of *S. typhi*. As a result, it has been concluded that CsgD is involved in a range of activities, including cellulose synthesis, bacterial pathogenicity, quorum sensing, and bacterial virulence.

**Conclusion:**

All identified targets in this study possess hydrophobic properties, and their cellular localization offered proof of a potent therapeutic target. Overall results of this study, drug target shortage in *S. typhi* is also spotlighted, and we believe that obtained result could be useful for the design and development of some potent anti-salmonella agents for typhoid fever in the future.

## 1 INTRODUCTION

*Salmonella* infection causes millions of deaths worldwide, necessitating the urgent development of effective therapeutic targets to treat and manage the set of diseases [1]. *Salmonella* infection manifests itself in two ways: typhoid and non-typhoid. Typhoid fever is caused by the *Salmonella typhi*, although non-typhoid illnesses are caused by the *Salmonella typhimurium*, *Salmonella enteritidis*, and other bacteria. Antibiotics have previously been used to treat both typhoid and non-typhoid *Salmonella* infections. On the other hand, antibiotics do not appear to be effective nowadays due to the emergence of antibiotic resistance that causes an increase in *Salmonella* infections [2]. As a result, the majority of second, third, and fourth-generation antibiotics are less effective, and the majority of people are unresponsive against available drugs [3]. Antibiotic resistance or drug failure is not the root problem in *Salmonella* infections. As *Salmonella* cells can form aggregation and clamping on the biological and non-biological surface with the formation of a special type of the protection covering layer, and that covering is known as a biofilm [4]. Biofilm protects from a variety of stress, including low oxygen, antibiotic assault, and the immune system of the host. The biofilm outer layer matrix, efflux pump, biological inert cell development (Persister cells), and quorum sensing all have a unique association in the biofilm with specific factors that play a vital role in antibiotic resistance [5]. As a result, a majority of antibiotics are resistant to *S. typhi* infection. Carbohydrates, proteins, and lipids constitute the extracellular matrix. Curli, cellulose, biofilm-associated protein, lipopolysaccharides, O antigen, Vi polysaccharides, and extracellular DNA are all found in *S*. *typhi* biofilms [6]. Due to the complicated structure of the biofilm, antibiotic drug molecules are unable to penetrate the biofilm. The *S. typhi* serovar showed high similarity with *S. typhimurium* serovar; therefore, we used the *S. typhimurium* as a reference’s genome during the protein and gene selection and mining process. The *S. typhimurium* biofilm has cellulose as one of its key components in the extracellular matrix, and the BcsA gene is important for cellulose synthesis [7]. We used the *S. typhi* biofilm protein, which showed a high similarity with the *S. typhimurium* biofilm protein. Thus, we targeted the matrix components and proteins of *S. typhi* biofilm with the help of existing literature, protein-protein networks, and other computational operations in this study. Consequently, we constructed a well-organized protein network, which was responsible for biofilm formation in *S*. *typhi.* The study aimed to identify a cascade of target proteins in the *S. typhi* biofilm condition with the help of genomic data mining and protein-protein interaction analysis. These proteins might be used as a therapeutic target for preventing *S. typhi* biofilm development and curing typhoid infection.

## 2 MATERIALS AND METHODS

### 2.1 Data Mining for Identification of the Biofilm Protein

Biofilm-related proteins had gathered from the NCBI database (https://www.ncbi.nlm.nih.gov/), UniProtKB (https://www.uniprot.org/), PDB (https://www.rcsb.org/), and KEGG databases for this study. In the creation and maturation of *S. typhi* biofilms, a total of 115 proteins were involved (https://www.ncbi.nlm.nih.gov/ipg/?term=SALMONELLA%20TYPHI%20BIOFILM). Through particular routes, all of the above proteins have various activities in biofilm like formation process, maturation process, quorum sensing, *etc*. However, this study only focused on central extracellular matrix proteins that were linked to other accessory biofilm proteins and processes. This study finds the role of a specific protein in *S. typhi* biofilm through the KEGG pathway analysis and NCBI PubMed available literature.

### 2.2 Selection of the Non-homologous Proteins

Only specific proteins were excluded from experiments that had shown a relationship with human proteins through NCBI Protein-Protein blast. As homologous proteins did not work as a drug targets; therefore, only non-homologous proteins were kept which have a specific role in the biofilm formation. Some proteins showed a high similarity with *S. typhimurium* Biofilm protein based on NCBI Pub Med literature [8]. A total of 15 proteins were found that play a crucial role in *S. typhi* Biofilm and few proteins were identical to those found in *S. typhimurium* Biofilm. However, only three proteins had the greatest resemblance to *S. typhimurium* Biofilm proteins based on the protein-protein blast. The KEGG pathway confirmed these proteins’ functional roles [9].

### 2.3 Cellular Localization of Target Protein

The Cello2go tool was used to determine the cellular location of all target proteins (http://cello.life.nctu.edu.tw/cello2go/). Cello2go is a tool that uses homology and blast searches to offer extensive information on protein subcellular localization [10]. Cello2go predicted the subcellular localization in archaea, gram-positive bacteria, gram-negative bacteria, and eukaryotes. Using Cell2go, we discovered the locations of all 15-target proteins that could be used to combat *S. typhi* Biofilm infections.

### 2.4 Perform Protein-protein Interaction

Protein-protein interactions analysis was performed by a computational tool STRING (https://string-db.org/cgi/input?sessionId=blESZ8flQx1x&input_page_show_search=on). The STRING has computational and experimental protein data to identify the protein-protein interactions. It covers the primary and secondary protein interactions with query proteins. Only selected protein as a query protein shows the interaction with other protein molecules, and only protein-protein interaction considered the confidence score ≥ 0.7 Hence STRING provided help to identify the specific relationship between one protein to another protein based on interaction [11].

### 2.5 Analysis of the Protein-protein Interaction

The protein-protein interaction network was analyzed using the Cytoscape 3.9.1 tool (https://cytoscape.org/) with the default parameters. The computational integrated network was analyzed, and Cytoscape presented the complex information in a clear and organized manner. The tool provided specific gene interactions and demonstrated the involvement of multiple genes with their appropriate sources of interaction [12].

### 2.6 Design the Pathogen-specific Protein Link Network

STRING provided the protein interaction network for query protein individually. Consequently, we designed the new protein network based on the functional characteristics of a gene or protein and the relationship between the two or more proteins, which are responsible for biofilm formation and regulations. These protein link networks had reflected as a hypothetical pathway for biofilm formations. However, this pathway already exists in *S. typhimurium* and is responsible for biofilm formations [13].

### 2.7 Prediction of Class and Family of the Target Protein

Target protein family predictions were carried out using the InterPro tool (https://www.ebi.ac.uk/interpro/). The InterPro database organizes protein sequences into families using an integrated categorization system. InterPro is a UniProt knowledgebase utility that combines 14 specialized member databases. Each InterPro member database specialized in a distinct field, and together they mainly provide complementary degrees of protein categorization, spanning from broad-level to fine-grained [14]. We used the InterPro method to identify all target protein families, and this prediction is highly useful in the field of medication discovery in fighting against the *S. typhi* biofilm-mediated infections.

### 2.8 Assessment of Drug Targeting Potential

**Fig. (1):**
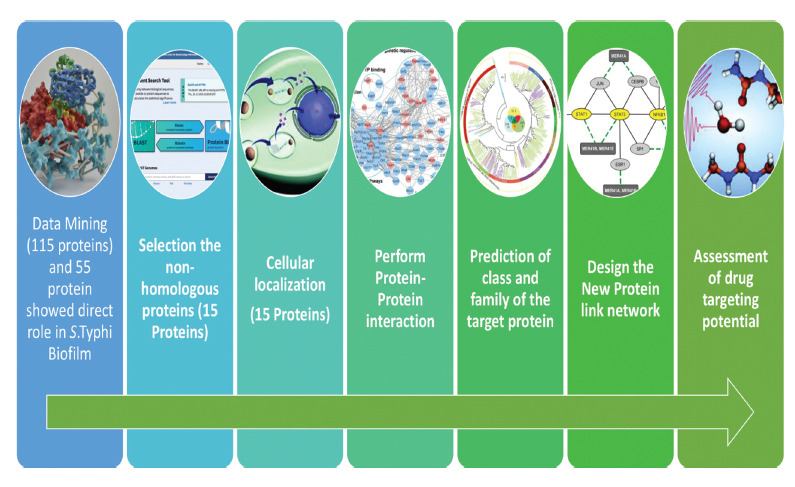
Work pipeline to perform the computational operation to the identification of potential target protein in *S. typhi* biofilm.

A therapeutic target's potential is governed by several factors, one of which is hydrophobicity. Due to their hydrophobicity, proteins have a direct association with electrostatic interaction and hydrogen-bonding networks. For the proper action of drug molecules, electrostatic interaction and hydrogen bonding networks are responsible for drug-ligand binding. It functioned as a protein calculator and calculated physicochemical properties such as hydrophobicity percentage. Using the Prot-pi program (https://www.protpi.ch/), we calculated the hydrophobicity percentage of all target proteins, which aids in selecting promising protein therapeutic targets (Fig. **1**).

## 3 RESULTS

The present study aimed to identify novel targets in *S. typhi* biofilm, and the obtained result was quite interesting, which was explained below pointwise.

### 3.1 Data Mining for Identification of the Biofilm Protein

From the NCBI database, UniProtKB, PDB, and KEGG databases, we shortlisted 115 proteins that had linked with *S. typhi* biofilm. Various proteins had shown to be involved in the biofilm of *S. typhi*; however, our inclusion criteria was based on both; direct and indirect relationships in the biofilm formation like adhesion phase relationship, maturation phase relationship, and dispersion phase relationship. Out of 115 proteins, we ruled out 60 proteins that have no direct connection to the *S. typhi* Biofilm. Only 55 proteins had shown a direct role in *S. typhi* biofilm formation. In this study, we scrutinized all proteins showing the biofilm link through the KEGG pathway.

### 3.2 Selection of the Non-homologous Proteins

Based on sequence similarity, we further inquired whether 55 proteins had any resemblance to human proteins. As a result, we utilized the NCBI BlastP tool to identify protein similarity, and 40 proteins had excluded from this experiment due to the human-like or homologous features. Consequently, we wanted to know whether the 15 non-homologous protein was similar to the *S. typhimurium* biofilm protein. Because the *S. typhimurium* serovar is well-researched in the field of biofilms. Phylogenetic tree-based analysis of *S. typhimurium* revealed 99 percent similarity [15]. We used the NCBI protein-protein blast method and kept the E value under the 7e-16. Only 15 non-homologous proteins had meted this criterion, and three biofilm proteins, such as CsgD, BcsA, and AdrA, had shown to have a 95 percent resemblance to *S. typhimurium* biofilm proteins (Table **1**).

### 3.3 Cellular Localization of Target Protein

The importance of cellular localization stems from the need to fully comprehend protein behaviour and function at specific cellular locations, such as the variations between protein production and protein action sites, also known as protein transportations [16]. By using Cello2go computational methods, we were able to determine the cellular localization of all 15-target proteins with default parameters (Table **1**), and three biofilm proteins (CsgD, BcsA, and AdrA) were found in the cytoplasmic and Inner Membrane regions, respectively. Our cello2go results had clearly shown that most biofilm proteins were presented in cytoplasmic areas and inner membrane areas (Table **1**). Hence, this area may work as a biofilm-controlling zone at cellular levels.

### 3.4 Protein-protein Interaction

**Fig. (2):**
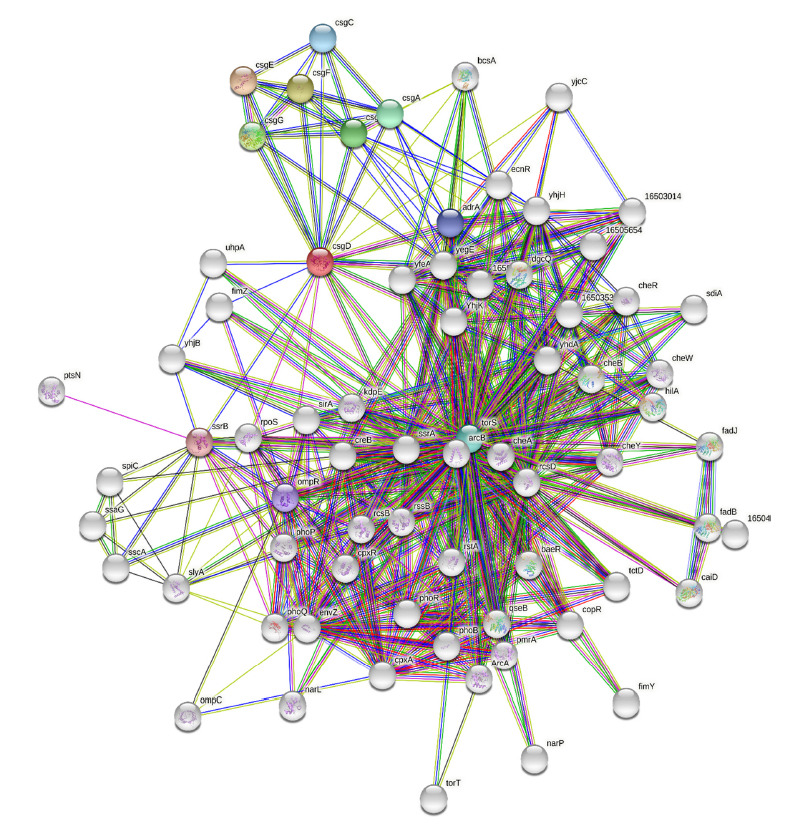
CsgD Protein-protein network showing interaction with other essential targets of *S. typhi*: CsgD protein linked with the other protein like bcsA, adrA, and Csg family protein and collectively form a cascade for inhibition of *S. typhi* biofilm.

**Fig. (3):**
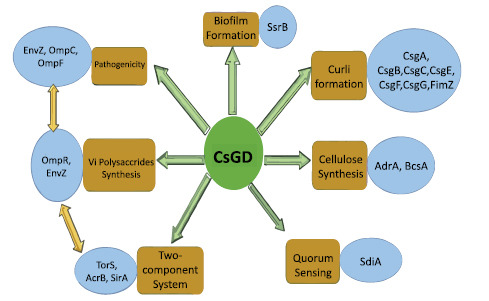
CsgD protein link network: CsgD protein linked with other phenomena like curli formation, cellulose synthesis, quorum sensing, two-component system, vi polysaccharides, and pathogenicity, which is essential for biofilm formation in *S. typhi.*

With all 15 target proteins, we used the STRING Tool to investigate protein-protein interactions because one protein interacts with another protein to form a protein network, and one protein can play a key part in several pathways. Only the CsgD protein had shown to be strongly interconnected with the other 14 proteins, but no additional proteins were found in the 14 reaming proteins (Fig. **2**). Due to this, we chose to study protein interaction with CsgD. The CsgD protein had primary contact with proteins CsgA, CsgB, CsgC, CsgE, CsgF, CsgG, OmpR, TorS, SsrB, and AdrA with a significant confidence score of 0.7, and a secondary interacted with BcsA, ArcB, SdiA, FimZ, and others. Based on UniProtKB and KEGG pathway analysis, these proteins had found to be involved in curli formations (Csg family, FimZ), Vi polysaccharides synthesis (OmpR), Two-component system (TorS, ArcB), biofilm formation (SsrB), diguanylate cyclase activity (AdrA), cellulose synthesis (BcsA), and quorum sensing (SdiA) in *S. typhi.* These proteins similarly affected other *Salmonella* species, such as *S. typhimurium*. According to one research, the Csg family is involved in the curli creation and assembly process, which is critical for *Salmonella b*iofilm adherence [17]. Other researchers believed that BcsA was involved in cellulose synthesis, ArcB was involved in the two-component system, SdiA had involved in the quorum-sensing process, and FimZ had involved in the fimbrial or curli formation process in *Salmonella* species [9, 18-20]. According to STRING findings, the CsgD protein served as a central protein because it was involved in a variety of pathways involving direct and indirect protein interactions, including curli formation and assembly, cellulose synthesis, the two-component system, biofilm formation, quorum sensing, and pathogenicity (Figs. **2** and **3**).

### 3.5 Analysis of the Protein-protein Interactions

**Fig. (4):**
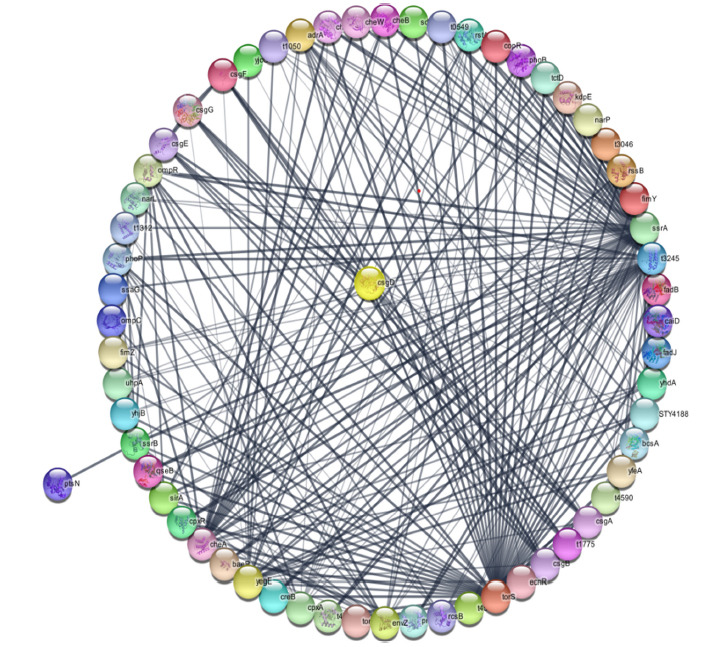
Protein-protein interaction analysis: CsgD worked as a central protein.

After analyzing protein-protein interactions using the STRING database, we utilized the Cytoscape tool to visualize the resulting network systematically. The network comprised 15 selected protein interactions, extracted from various data sources, including KEGG, STRING cluster, Smart domain, InterPro, Pfam, and GO Biological Process. Within this network, we identified four proteins that directly participate in biofilm formation, namely Adr, CsgG, CsgF, and CsgE. Additionally, several proteins were found to be involved in the assembly of curli and flagella, including CsgD, CsgB, CsgA, and CsgE. These proteins also contribute to biological adhesion activity with Adra proteins. A few proteins in the network, such as CsgD, SsrB, SdiA, OmpR, and FimZ, played significant roles in DNA binding and transcriptional regulation. Specifically, CsgD binds to specific regions of DNA to regulate transcription. Furthermore, several proteins played key roles in the bacterial two-component system, which is necessary for motility, virulence, and nutrient acquisition. These proteins include SdiA, OmpR, SsrB, FimZ, EnvZ, and Tors. Overall, our selected proteins played crucial roles in various pathways and were interconnected. In this network, every protein contacted each other, and CsgD played a central role due to the connectivity with other remaining proteins. Therefore, all 15 proteins could potentially be targeted for therapeutic purposes in *S. typhi* biofilm infections (Fig. **4**).

### 3.6 Design the Pathogen-specific Protein Link Network

**Fig. (5):**
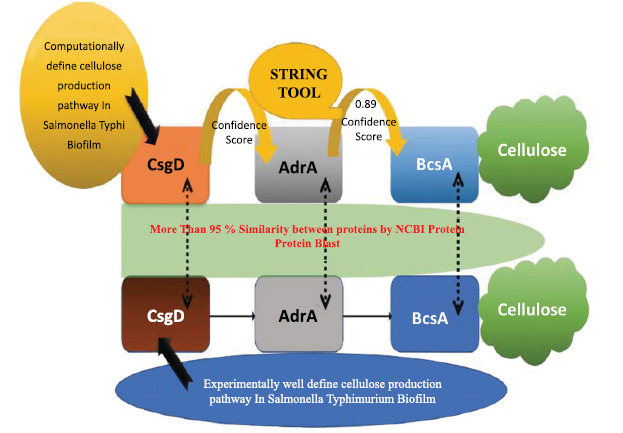
Involvement of CsgD in cellulose production pathway of *Salmonella typhi* based on the reference organism *S. typhimurium.*

In the extracellular matrix of *S. typhi* biofilms, cellulose is a significant component. Based on UniProtKB data and data mining, our research clearly showed that the BcsA protein played a crucial part in the cellulose synthesis process. A study suggests that the AdrA protein performed the role in cellulose synthesis in *S. typhimurium* [21]. According to STRING data, the AdrA protein in *S. typhi* is firmly related to the BcsA protein with a 0.89 confidence score, while the CsgD protein interacted with AdrA with a 0.76 confidence score. As a result, CsgD had a primary interaction with the AdrA protein and secondary interaction with the BcsA protein, which played a key role in cellulose production in biofilms (Figs. **3** and **5**). Another previous study clearly showed that CsgD controls cellulose production through AdrA and BcsA protein in *S. typhimurium* [13]. STRING, blast, and KEGG analysis confirmed that these phenomena occurred in *S. typhi.* During the same STRING analysis tool, CsgD revealed links to Csg family proteins with confidence scores ranging from 0.8 to 0.89, indicating that they played a key part in the curli formation process. The previous study suggests that curli and cellulose were an important part of the *S. typhimurium* Biofilm whose expression is controlled by CsgD proteins [22]. Our protein network had evidence that CsgD works as a central biofilm protein in *S. typhi* (Fig. **2**).

### 3.7 Prediction of Class and Family of the Target Protein

Our next result is the identification of the target protein done by the InterPro tool. Previous tools defined the target predictions and pathways but not delivering the protein family information. Csg proteins belong to the curli production family, secretion system family, and transport component family. BcsA protein had a major role in cellulose production, which belonged to the cellulose synthase family and torS, arcB had shown a relationship with signal transduction Histidine Kinase family (Table **1**). It showed a very important characteristic for drug discovery because it provides common information about proteins based on only family predictions.

### 3.8 Assessment of Drug Targeting Potential

Hydrophobicity analysis was included as the top characteristic of measuring the drug target potential because it provides information about ligand-target binding capacity information. We analyzed the total hydrophobicity percentage based on every amino acid characteristic percentage with the help of the Prot-pi program tool with the set default parameters. Protein contains four types of amino acids, mainly hydrophobic, hydrophilic, basic, and acidic, based on amino acid sequences. The hydrophobicity percentage of our all-target proteins ranged from 39% to 53% was a respectable proportion for therapeutic target potential (Table **1**). The maximal hydrophobicity of the AdrA protein was 54.05%, the BcsA protein was 53.31%, and the CsgD protein was 41.83%. Our results indicate that all target proteins had good hydrophobicity characteristics, which provided more evidence for work as a good drug target in *S. typhi.*

## 4 DISCUSSION

*S. typhi* biofilm infection is a growing health concern, and it showed a co-relationship with other serious diseases such as a gallbladder cancer [23, 24], because *S. typhi* biofilm releases a specific type of toxins and proteins, which is responsible for DNA damage and alter the metabolic pathways. Biofilm provides a supportive environment for the survival of the bacteria during the stress conditions such as a exposure to antibiotic stress, nutritional depletion and host immune response, *etc*., and causes serious health issues [25, 26]. Hence, It is vitally necessary to control the *S. typhi* biofilm infection. Therefore, our study was focused on the identification of potential targets in *S. typhi* biofilm so that target-based therapeutics are designed in the future. We identified 15 target proteins that are involved in biofilm formation, maturation, and pathogenicity pathways. Only the CsgD protein showed maximum interconnections among reaming 14 proteins. The CsgD protein showed a well-defining role as the master biofilm regulatory protein in *S. typhimurium* [27]. CsgD protein of *S. typhi* had shown 99 percent similarity with *S. typhimurium* CsgD protein. Our protein-protein interaction study indicates that CsgD played a significant role in cellulose production by interacting with the BcsA protein *via* the AdrA protein. This finding was supported by UniProtKB, which had established the crucial role of BcsA in cellulose production. Notably, our results revealed a high confidence score of 0.76 between CsgD and AdrA and a score of 0.89 between AdrA and BcsA, suggesting a potential interaction between these proteins. Consequently, CsgD, AdrA, and BcsA formed an interaction complex for cellulose production in *S. typhi* biofilm. Moreover, we found that SdiA played a significant role in the quorum-sensing process of *S. typhimurium* [28]. The quorum sensing process allows bacteria to communicate with each other and coordinate their survival strategies against antibiotic attacks. Therefore, disrupting the communication network is essential to control biofilm infections. *S. typhi* SdiA protein has shown over 98% similarity with *S. typhimurium* SdiA protein (based on protein-protein blast analysis). The protein-protein interaction study further revealed that SdiA interacted with CsgD in *S. typhi* through the SsrA and SsrB proteins. While the exact pathway remains unknown in *S. typhi*, however, interaction showed a significant confidence score of 0.708 between the CsgD protein and SsrB. Based on our findings, it appeared that CsgD was linked with SdiA, suggesting that CsgD played a role in the quorum-sensing process of *S. typhi*. This is a significant finding, as it highlights the potential of SdiA as a drug target for controlling biofilm infections. Indeed, our computational approach identified SdiA as an attractive candidate for further investigation as a potential therapeutic target. Various studies suggest that curli shows as a key component of *S. typhi* biofilm and Csg family protein are showing involvement in the formation and maturation of the curli, which provides the bacterial attachment with the surface [17, 29]. Results provided compelling evidence of protein-protein interactions between the members of the Csg family (A, B, C, D, E, F, G). Notably, CsgD has been shown a link with other members of the Csg family, including CsgA (with a confidence score of 0.87), CsgB (0.87), CsgC (0.82), CsgE (0.98), CsgF (0.98), and CsgG (0.93). These results suggest that there is a robust interaction between CsgD and other Csg family members, highlighting the importance of CsgD in the formation and maturation of curli and bacterial attachment to surfaces in *S. typhi* biofilm. Therefore, targeting the Csg family proteins could be a promising approach for designing drugs against the *S. typhi* biofilm. Notably, *S. typhi* possesses certain distinct characteristics that distinguish it from other pathogens, such as the Vi antigen and *Salmonella* pathogenicity island, which provide enhanced survival abilities within the host [30]. In this study, CsgD showed a direct relation with other bacterial proteins like ompR, envZ, torS and ompC, ompF, and acrB, which played a role in bacterial pathogenicity, Vi polysaccharides expression, *etc*.

## CONCLUSION

Most of the available therapies are not showing effective results against the *S. typhi* biofilm. Identifying new drug target proteins is urgently required; however, a target shortage is experienced in this field. Our study deciphered some potential druggable targets against the *S. typhi* biofilm condition. Based on the computational approach, all identified targets have an important role in *S. typhi* biofilm establishment, and these proteins have a good drug target potential. CsgD protein has a significant relationship with the other 14 target proteins of *S. typhi,* and they all played the regulatory role for *S. typhi* biofilm (Table **1**, Figs. **2-4**). Our study provided the idea about the strong druggable target against *S. typhi* biofilm, and it requires more experimental study to validate the information. The Csg family protein and other concerned proteins could work as strong targets that need further validation. Additionally, they could prove to be a major player in support of promising targets in *S. typhi* biofilm. The entire process of drug development warrants massive time, money, and resources to screen out ligands against the potent target. Therefore, before *in vitro* and *in vivo* experimentation, *in silico* analysis plays a great role in identifying essential targets. Although more detailed *in vitro* and *in vivo* research is required, however, this study provides a base for target identification and further exploration in the discovery and development of antibiofilm agents.

## Figures and Tables

**Table 1:** List of *Salmonella typhi* biofilm target proteins and their *in silico* characterization to know localization, classification, and other details.

**S. No.**	**Target Protein**	**UniProt ID/ Status**	**Detail of Protein**	**Localization of ** **Target Protein**	**Class/Family of the Target Protein**
1	CsgD	Q8Z7M4/ Unreviewed	Work as biofilm regulatory protein	Cytoplasmic	None
2	CsgA	P0A1E6/ Reviewed	Major curlin subunit	Extracellular	None
3	CsgB	Q8Z7M3/ Reviewed	Minor curlin subunit	Extracellular	None
4	CsgC	Q8Z7M2/ Unreviewed	Curli assembly protein	Inner membrane	Curli production protein
5	CsgE	P0A201/ Reviewed	Curli production assembly	Cytoplasmic	Curli assembly protein
6	CsgF	P0A203/ Reviewed	Curli production assembly	Extracellular	Type VIII secretion system
7	CsgG	P0A205/ Reviewed	Curli production assembly	Outer membrane	Transport component
8	AdrA	Q8Z8Z3/ Unreviewed	Diguanylate cyclase activity	Inner membrane	None
9	BcsA	Q8Z291/ Reviewed	Cellulose synthase catalytic subunit	Inner membrane	Cellulose synthase
10	TorS	Q8Z2M6/ Unreviewed	Histidine kinase	Inner membrane	Signal transduction histidine kinase
11	ArcB	Q8Z3F8/ Unreviewed	Aerobic respiration control sensor protein	Inner membrane	Singal transduction histidine kinase
12	FimZ	Q8Z8P0/ Unreviewed	Fimbria biosynthesis transcriptional regulator	Cytoplasmic	None
13	SSrB	Q8XFU4/ Unreviewed	DNA-binding response regulator	Cytoplasmic	None
14	SdiA	Q8Z5T1/ Unreviewed	Cell-division regulatory protein	Cytoplasmic	None
15	Ompr	P0AA20/ Reviewed	DNA-binding dual transcriptional regulator OmpR	Cytoplasmic	Transcriptional regulatory protein

## Data Availability

The authors confirm that the data supporting the findings of this research are available within the article.

## LIST OF ABBREVIATIONS

KEGG: Kyoto Encyclopedia of Genes and Genomes
NCBI: National Center for Biotechnology Information
PDB: Protein Data Bank

## References

[1] Wilairatana P., Mala W., Klangbud W.K., Kotepui K.U., Rattaprasert P., Kotepui M. (2021). Prevalence, probability, and outcomes of typhoidal/non-typhoidal Salmonella and malaria co-infection among febrile patients: A systematic review and meta-analysis.. Sci. Rep..

[2] Smith S.I., Seriki A., Ajayi A. (2016). Typhoidal and non-typhoidal Salmonella infections in Africa.. Eur. J. Clin. Microbiol. Infect. Dis..

[3] Karkey A., Thwaites G.E., Baker S. (2018). The evolution of antimicrobial resistance in Salmonella typhi.. Curr. Opin. Gastroenterol..

[4] Harrell J.E., Hahn M.M., D’Souza S.J., Vasicek E.M., Sandala J.L., Gunn J.S., McLachlan J.B. (2021). Salmonella biofilm formation, chronic infection, and immunity within the intestine and hepatobiliary tract.. Front. Cell. Infect. Microbiol..

[5] Høiby N., Bjarnsholt T., Givskov M., Molin S., Ciofu O. (2010). Antibiotic resistance of bacterial biofilms.. Int. J. Antimicrob. Agents.

[6] Thajuddin D.P.E-D.D.E-N. (2016). Biofilm Formation of Salmonella..

[7] Chandra M., Thakur S., Narang D., Kaur G., Sharma N.S. (2017). Evaluation of Salmonella typhimurium biofilm against some antibiotics.. Indian J. Exp. Biol..

[8] Sabbagh S.C., Forest C.G., Lepage C., Leclerc J.M., Daigle F. (2010). So similar, yet so different: uncovering distinctive features in the genomes of Salmonella enterica serovars typhimurium and Typhi.. FEMS Microbiol. Lett..

[9] Gill M.A., Rafique M.W., Manan T., Slaeem S., Römling U., Matin A., Ahmad I. (2018). The cellulose synthase BcsA plays a role in interactions of Salmonella typhimurium with Acanthamoeba castellanii genotype T4.. Parasitol. Res..

[10] Yu C.S., Cheng C.W., Su W.C., Chang K.C., Huang S.W., Hwang J.K., Lu C.H. (2014). CELLO2GO: A web server for protein subCELlular LOcalization prediction with functional gene ontology annotation.. PLoS One.

[11] Szklarczyk D., Franceschini A., Wyder S., Forslund K., Heller D., Huerta-Cepas J., Simonovic M., Roth A., Santos A., Tsafou K.P., Kuhn M., Bork P., Jensen L.J., von Mering C. (2015). STRING v10: Protein–protein interaction networks, integrated over the tree of life.. Nucleic Acids Res..

[12] Chen Q., Xia S., Sui H., Shi X., Huang B., Wang T. (2022). Identification of hub genes associated with COVID-19 and idiopathic pulmonary fibrosis by integrated bioinformatics analysis.. PLoS One.

[13] Liu Z., Niu H., Wu S., Huang R. (2014). CsgD regulatory network in a bacterial trait-altering biofilm formation.. Emerg. Microbes Infect..

[14] Hunter S., Apweiler R., Attwood T.K., Bairoch A., Bateman A., Binns D., Bork P., Das U., Daugherty L., Duquenne L., Finn R.D., Gough J., Haft D., Hulo N., Kahn D., Kelly E., Laugraud A., Letunic I., Lonsdale D., Lopez R., Madera M., Maslen J., McAnulla C., McDowall J., Mistry J., Mitchell A., Mulder N., Natale D., Orengo C., Quinn A.F., Selengut J.D., Sigrist C.J.A., Thimma M., Thomas P.D., Valentin F., Wilson D., Wu C.H., Yeats C. (2009). InterPro:The integrative protein signature database. Nucleic Acids Res.

[15] Chang H.R., Loo L.H., Jeyaseelan K., Earnest L., Stackebrandt E. (1997). Phylogenetic relationships of Salmonella typhi and Salmonella typhimurium based on 16S rRNA sequence analysis.. Int. J. Syst. Bacteriol..

[16] Dönnes P., Höglund A. (2004). Predicting protein subcellular localization: Past, present, and future.. Genomics Proteomics Bioinformatics.

[17] González J.F., Tucker L., Fitch J., Wetzel A., White P., Gunn J.S. (2019). Human bile-mediated regulation of salmonella curli fimbriae.. J. Bacteriol..

[18] Pardo-Esté C., Hidalgo A.A., Aguirre C., Inostroza A., Briones A.C., Cabezas C.E., Castro-Severyn J., Fuentes J.A., Opazo C.M., Riedel C.A., Otero C., Pacheco R., Valvano M.A., Saavedra C.P. (2019). Correction: The ArcAB two-component regulatory system promotes resistance to reactive oxygen species and systemic infection by Salmonella typhimurium.. PLoS One.

[19] Sholpan A., Lamas A., Cepeda A., Franco C.M. (2021). Salmonella spp. quorum sensing: an overview from environmental persistence to host cell invasion.. AIMS Microbiol..

[20] Kolenda R., Ugorski M., Grzymajlo K. (2019). Everything you always wanted to know about Salmonella type 1 fimbriae, but were afraid to ask.. Front. Microbiol..

[21] García B., Latasa C., Solano C., Portillo F.G., Gamazo C., Lasa I. (2004). Role of the GGDEF protein family in Salmonella cellulose biosynthesis and biofilm formation.. Mol. Microbiol..

[22] Grantcharova N., Peters V., Monteiro C., Zakikhany K., Römling U. (2010). Bistable expression of CsgD in biofilm development of Salmonella enterica serovar typhimurium.. J. Bacteriol..

[23] Upadhayay A., Pal D., Kumar A. (2022). Salmonella typhi induced oncogenesis in gallbladder cancer: Co-relation and progression.. Adv Cancer Biol: Metastasis..

[24] Upadhyay A., Pal D., Kumar A. (2023). Substantial relation between the bacterial biofilm and oncogenesis progression in host.. Microb. Pathog..

[25] Upadhyay A., Pal D., Kumar A. (2023). Combinatorial enzyme therapy: A promising neoteric approach for bacterial biofilm disruption.. Process Biochem..

[26] Upadhayay A., Ling J., Pal D., Xie Y., Ping F.F., Kumar A. (2023). Resistance-proof antimicrobial drug discovery to combat global antimicrobial resistance threat.. Drug Resist. Updat..

[27] Wen Y., Ouyang Z., Devreese B., He W., Shao Y., Lu W., Zheng F. (2017). Crystal structure of master biofilm regulator CsgD regulatory domain reveals an atypical receiver domain.. Protein Sci..

[28] Ahmer B.M.M., van Reeuwijk J., Timmers C.D., Valentine P.J., Heffron F. (1998). Salmonella typhimurium encodes an SdiA homolog, a putative quorum sensor of the LuxR family, that regulates genes on the virulence plasmid.. J. Bacteriol..

[29] Jonas K., Tomenius H., Kader A., Normark S., Römling U., Belova L.M., Melefors Ö. (2007). Roles of curli, cellulose and BapA in Salmonella biofilm morphology studied by atomic force microscopy.. BMC Microbiol..

[30] Pickard D., Li J., Roberts M., Maskell D., Hone D., Levine M., Dougan G., Chatfield S. (1994). Characterization of defined ompR mutants of Salmonella typhi: OmpR is involved in the regulation of Vi polysaccharide expression.. Infect. Immun..

